# Diacylglycerol Kinases in Signal Transduction

**DOI:** 10.3390/ijms23158423

**Published:** 2022-07-29

**Authors:** Sara Centonze, Gianluca Baldanzi

**Affiliations:** 1 Department of Translational Medicine, University of Piemonte Orientale, 28100 Novara, Italy; sara.centonze@uniupo.it; 2 Center for Translational Research on Allergic and Autoimmune Diseases (CAAD), University of Piemonte Orientale, 28100 Novara, Italy

In recent years, the significant research efforts put into the clarification of the PI3K/AKT/mTOR pathway resulted in the approval of the first targeted therapies based on lipid kinase inhibitors [1]. This increased attention toward similar pathways, with increasing interest in lipid signaling and specifically in lipid kinases. Among them, diacylglycerol kinases (DGKs) are of particular interest for their proven double role as negative modulators of the immune response and drivers of cancer cell growth [2].

By including both research articles and reviews, this special issue aimed to capture the state of art of the research on the DGK role in signaling and in phospholipid metabolism. The main focus was the progressive advancement in the understanding of DGK regulation and of their biological functions in different cellular contexts, as well as their contribution to disease development. Of particular interest were the recent efforts aiming to capitalize the available knowledge on therapeutic advancements using pharmacological inhibitors and genetic tools, despite the still incomplete knowledge of DGK structures.

Fazio et al. explored the fascinating level of complexity of DGKs position in lipid signaling, within the context of phosphatidylinositols signaling and cancer-associated cellular mechanisms, such as cell growth, proliferation, and migration [3]. As they highlighted, both the diacylglycerol (DAG) substrate and the phosphatidic acid (PA) product result from the activity of other regulated enzymes, generating a multiplicity of small intracellular pools tuned by DGK. They concluded that more attention is required on the still partially characterized link between those localized lipid signals and the oncogenic properties of some DGK isoforms.

Sim et al. discussed how, to coordinate so many functions, a network of regulatory signals finely tunes DGK activity and localization [4]. Those signals are mediated by isoform specific regulatory domains and by an increasing number of interactors, which precisely control the DGK activity in each cell compartment. The local DAG and PA pools in turn regulate the activity and localization of a constantly rising number of effectors, justifying the pleiotropic effects that DGKs have on the major signaling pathways. Finally, the authors gave us some insights regarding the possibility of modulating PA synthesis using DGK inhibitors in combination with inhibitors of other PA-producing enzymes, in an attempt to improve the current therapeutic strategies against cancer.

The DGK-mediated PA production at the membrane converts the small neutral DAG to a cone shaped negatively charged PA, thus regulating membrane curvature and providing negative charges that bind protein regions with stretches of positively charged amino acids of adequate shape. In their work, Sakane et al. reviewed recent studies on the PA species produced by each DGK isozyme, with particular attention on those not derived from phosphatidylinositol’s turnover and to their ability to bind selectively to different effectors [5]. Indeed, using specific DGK-generated phosphatidic acid species, the authors found several new PA binding proteins characterized by distinct selectivity for specific acyl chains. This contributed to increasing the list of PA-binding proteins, which today comprises protein/lipid kinases, protein/lipid phosphatases, phospholipases, phosphodiesterases, G-proteins, and G-protein regulators. The investigation of PA effectors allowed the generation of specific probes to localize this lipid, but also suggests that these proteins may be part of a complex signaling network which controls a wide range of both biological and pathological events.

Despite the translational interest in DGKs, the exploration of DGKs’ biological functions has focused mainly on the immune and nervous system, while translational approaches have concentrated on cell transformation and cancer. This is just the tip of the iceberg, as evidenced by the work of Velnati et al. on the function of the DGKα isoform in X-linked lymphoproliferative disease type 1, a genetic disorder where X-linked mutations of the SAP adaptor cause excessive DGKα activity and reduce DAG signaling. This perturbation of the DAG/PA balance contributes to the immune dysregulation typical of this disease, and specifically to reduced restimulation induced cell death, which makes patients exceedingly sensitive to EBV infection. These defects are compensated for in vitro and in animal models by DGKα inhibitors [6]. DGKs’ role in genetic diseases is a growing field of research, as illustrated by the observation that DGKk hypofunctionality is a key feature of fragile X syndrome [7], and DGKε loss of function is causative of hemolytic uremic syndrome [8].

Experimentally generated loss of function mice has been crucial for the identification of further DGK’s biological functions. In the brain, these studies indicated a precise, isoform-specific modulation of lipid homeostasis that has a crucial role in the context of synaptic plasticity. Tsumagari and colleagues generated Purkinje cell-specific DGKγ KO mice using the Cre-loxP recombination system to study the effect of DGK-mediated PKCγ regulation on motor coordination [9]. Their results demonstrated that, in DGKγ’s absence, the negative regulation of the calcium channel TRPC3 by PKCγ overactivation inhibits PKCα activity during long-term depression, resulting in motor dyscoordination. Thus, the authors clarified a new mechanism by which DGKγ, modulating PKCγ basal activity, is crucial for cerebellar long-term depression.

With this Special Issue, we brought together functional studies exploring DGKs’ biological functions [9], their involvement in genetic diseases [6], and effectors of DGK-generated PA [5], with studies on DGKs’ regulative role in signaling [3,4]. From different points of view the contributors evidenced a great translational potential in the DGK field. To be exploited, this potential awaits a better knowledge of DGKs’ direct effectors and for new pharmacological tools to explore their biological relevance. Indeed, the current DGK inhibitors are limited to molecules that are quite DGKα specific and are rarely used in vivo [10]. We expect rapid improvements in the DGK inhibitors field thanks to the recent advancements in computational tools such as AlphaFold, which are starting to provide good models of DGK structures for rational drug design.

We conclude by gratefully acknowledging all the authors for their contribution to this fast-developing research topic.
